# Autoimmune-Mediated Retinopathy in CXCR5-Deficient Mice as the Result of Age-Related Macular Degeneration Associated Proteins Accumulation

**DOI:** 10.3389/fimmu.2019.01903

**Published:** 2019-08-14

**Authors:** Anton Lennikov, Madhu Sudhana Saddala, Anthony Mukwaya, Shibo Tang, Hu Huang

**Affiliations:** ^1^Department of Ophthalmology, University of Missouri, Columbia, MO, United States; ^2^Johns Hopkins University School of Medicine, Baltimore, MD, United States; ^3^Department of Ophthalmology, Faculty of Health Sciences, Institute for Clinical and Experimental Medicine, Linköping University, Linköping, Sweden; ^4^Aier School of Ophthalmology, Aier Eye Institute, Central South University, Changsha, China

**Keywords:** age macular degeneration, AMD, CXCR5, CXCL13, autoantibody, CRYAA, CRYAB, amyloid beta

## Abstract

Previous research has shown that CXCR5^−/−^ mice develop retinal degeneration (RD) with age, a characteristic related to age macular degeneration (AMD). RD in these mice is not well-understood, and in this study, we sought to characterize further the RD phenotype and to gain mechanistic insights into the function of CXCR5 in the retina. CXCR5^−/−^ and WT control mice were used. Fundus images demonstrated a significant (*p* < 0.001) increase of hypo-pigmented spots in the retina of aged CXCR5^−/−^ mice compared with WT control mice. PAS staining indicated localization of deposits in the sub-retinal pigment epithelia (RPE) layer. AMD-associated proteins Cryab, amyloid beta, and C3d were detected within the RPE/sub-RPE tissues by immunofluorescence (IF). In addition, western blot analysis of COX-2, Arg1, and VEGF-a revealed an increase in the signaling of these molecules within the RPE/choroid complex. Transmission electron microscopy (TEM) indicated a drusen-like structure of sub-RPE deposits with an accumulation of vacuolated cellular debris. Loss of photoreceptors was detected by peanut lectin staining and was corroborated by a reduction in MAP2 signaling. Loss of blood-retinal barrier integrity was demonstrated by a reduction of ZO-1 expression. Inflammatory cells were detected in the sub-RPE space, with an increase in IBA-1 positive microglia cells on the surface of the RPE. Mass spectrometry analysis of CXCR5^−/−^ mouse RPE/choroid proteins extracts, separated by SDS-page and incubated with autologous serum, identified autoantibodies against AMD-associated proteins: Cryaa, Cryab, and Anxa2. *In vitro* evaluations in BV-2 cell culture indicated a significant increase in production of Arg-1 (*p* < 0.001) and COX-2 (*p* < 0.01) in the presence of anti-CXCR5 antibody when compared with Igg-treated control BV-2 cells stimulated with IL-4 and TNFα/IFNγ, respectively. Anti-CXCR5 antibody treatment without stimulating agents did not affect Arg-1 and COX-2 expression; this suggests that CXCR5 may have a regulatory role in microglia cells activation. These results indicate that with age, CXCR5^−/−^ mice develop RD characterized by microglia dysfunction, increased production of CXCL13 in the RPE progressive photoreceptor, neuronal loss, and sub-RPE deposition of cellular debris, resulting in the production of immunogenic proteins and autoimmune-mediated RD.

## Introduction

Age-related macular degeneration (AMD) is the leading cause of blindness in adults older than 65 years of age (1). It is a complex heterogeneous disease that first manifests in the macula with the appearance of pigmentary changes and subretinal deposits called drusen. Analysis of the composition of drusen deposits and surrounding retinal pigment epithelia (RPE) layer cells in humans with AMD identified amyloid beta (Aβ) (2), alpha b crystallin (CRYAB), and alpha a crystalline (CRYAA) (3, 4) as the most significant components of this pathology. AMD can progress as a “dry” degenerative form, leading to geographic atrophy (GA) of the retinal pigment epithelium (RPE), choriocapillaris, and photoreceptors (5). In some cases, AMD can develop as neovascular or a “wet” form mediated predominantly through VEGF signaling (6). To date, there has been no scientific consensus if the “dry” and “wet” forms of AMD are two stages of the same disease or if they are two separate pathologies (7). From a clinical perspective, pharmacological inhibition of VEGF-A is effective for the management of pathologic angiogenesis and vascular leakage in patients with “wet” AMD (8). However, no treatment exists for “dry” AMD.

In addition, rodent models of AMD, such as those used to investigate the complement factor pathways (e.g., cfh^−/−^ mice, C3a, and C5a receptor^−/−^ mice) (9), chemokine (Ccl2^−/−^ and Ccr2^−/−^ mice) (10), and oxidative stress (Sod1^−/−^ mice) (11)—among others—do not accurately mimic the pathophysiology of this disease (12). Previously, several mouse models were introduced to have “dry” AMD RD, including Ccl2 and Cx3cr1 knockout mice; these models demonstrated promising results, but later degenerative changes in the retina were found to be attributed predominantly to the single nucleotide polymorphism (SNP) rd8 mutation in the CRB1 gene, which is especially common in C57/BL6N animals. These models also failed to capture human-like AMD degenerative changes (13).

C-X-C motif chemokine receptor 5 (CXCR5)—a chemokine transmembrane receptor belonging to the CXC chemokine receptor family—plays an essential role in B cell activation through its ligand (C-X-C motif): ligand 13 (CXCL13). Initially identified in Burkitt lymphoma cells and various tumors (14, 15), recent studies have indicated that the CXCR5/CXCL13 signaling axis may play a significant role in the central nervous system, such as in pain transduction (16). Also, CXCL13–CXCR5 is associated with the infiltration of B cells into the brain during neonatal development (17).

The ablation of CXCR5 in mice resulted in neuronal immaturity and decreased neuronal proliferation (18). In zebrafish, CXCR5 mutations impair brain regeneration capacity (19). CXCL13 secreted by microglia is involved in the recruitment of CXCR5 expressing Th1, Th17, and B cells in multiple sclerosis (MS) (20). Furthermore, elevated levels of CXCR5 have been found in the aqueous humor of AMD patients (21).

Recently, we showed that aged CXCR5 knockout mice (CXCR5^−/−^) develop retinal degeneration (RD) (22) with pathophysiological changes, such as disruption of photoreceptors, upregulation of TNFα with the presence of apoptotic cells in the retina, and loss of ZO-1 indicative of impaired blood-retinal barrier function. These changes indicate that CXCR5 knockout mice are a potentially invaluable strain for AMD research. Here, we further characterize the previously observed phenotypic changes in CXCR5^−/−^ mice to understand better the pathophysiology of these changes in relation to AMD associated proteins and autoantibodies. In our work, we conducted robust screening of all the animals and the murine cells included in the study to ensure they were CRB1-RD8 mutation-free (Supplementary Figure 5). We did this to ensure that all the observed changes in the retina can be attributed to a lack of CXCR5.

## Results

### Fundus and Fluorescent Angiography Imaging

**Figure 1:**
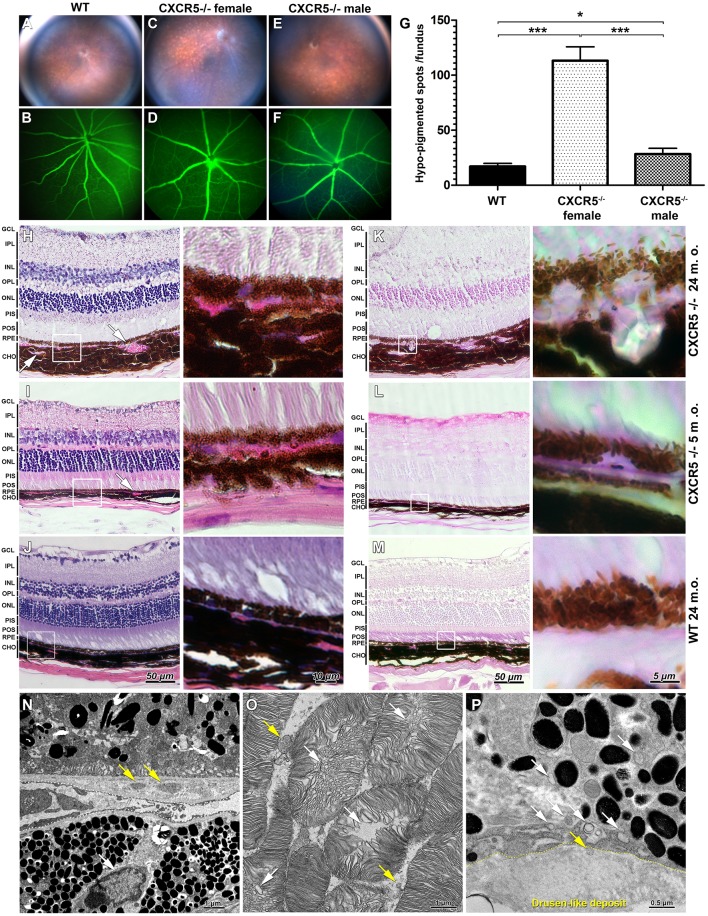
Fundus images and fluorescent angiography (FA) H&E, periodic acid Schiff (PAS) staining of CXCR5^−/−^ and age-matched WT controls as well as transmission electron microscopy (TEM) of aged CXCR5^−/−^ retinal pigment epithelia and retina. **(A,B)** Fundus images and fluorescent angiography of 24 m.o. female WT mouse; **(C**, **D)** 24 m.o. female CXCR5^−/−^ mouse; **(E**, **F)** 24 m.o. CXCR5^−/−^ male mouse; **(G)** Quantitative analysis of retinal hypo-pigmentation spots (*n* = 6). A one-way ANOVA test with Tukey multiple comparisons was used to determine statistical significance. *p < 0.05; ***p < 0.001. **(H)** H&E staining of 24 m.o. CXCR5^−/−^; **(I)** 5 m.o. CXCR5^−/−^; **(J)** 24 m.o. WT mice; **(K**–**M)** PAS staining of adjacent sections of the same eyes CXCR5^−/−^; Choroidal neovascularization is indicated by white arrows. The white square represents the enlarged RPE/choroid area of the respective image. Retinal layer designations: GCL, ganglion cell layer; IPL, inner plexiform layer; INL, inner nuclear layer; OPL, outer plexiform layer; ONL, outer nuclear layer; PIS, photoreceptors inner segments; POS, photoreceptors outer segments; RPE, retinal pigment epithelia; CHO, choroid. All histological samples were obtained from female mice. **(N)** Ultrastructural overview of retinal pigment epithelia, Bruch's membrane, and choroid of 24 m.o. female CXCR5^−/−^ mouse; basal linear deposits are indicated by yellow arrows; the white arrow indicates a lymphocyte within the choroid near Bruch's membrane; **(O)** Photoreceptors—disruption of photoreceptors indicated by white arrows, and yellow arrows indicate that fragmented debris of the photoreceptors cells was present in intracellular space. **(P)** Sub-RPE drusen-like deposits ultrastructure—the yellow dotted line indicates the border of the deposit; the white arrows indicate vesicular structures; the yellow arrow indicates the vesicle entering the deposit.

Representative fundus and fluorescent angiography (FA) images of aged C57/BL6J (WT) female (Figures 1A,B), age-matched CXCR5^−/−^ female (Figures 1C,D), and CXCR5^−/−^ male mice (Figures 1E,F). The CXCR5^−/−^ animals demonstrated an increase in the number of hypo-pigmentation spots in the retina (Figure 1C). No difference between the CXCR5^−/−^ (Figures 1D,F) and WT control mice (Figure 1B) was observed in the structure of retinal vasculature, which also did not have any leakage. Quantitative analysis of pigmentation demonstrated significant (*p* < 0.001) increase in hypo-pigmentation spots in CXCR5^−/−^ mice compared to the control mice. The abnormalities observed by fundus imaging were pronounced more in the females compared to the male CXCR5^−/−^ mice of the same age (*p* < 0.001). Although male CXCR5^−/−^ mice demonstrated fewer hypo-pigmented spots than female CXCR5^−/−^ mice (*p* < 0.001), the number of the hypo-pigmented spots was still significantly higher in male CXCR5^−/−^ mice than in the WT controls (*p* < 0.05) (Figure 1G).

### Histological and TEM Findings in the Retina and Sub-RPE Space

Hematoxylin and eosin (H&E) staining revealed increased choroidal neovascularization and slightly eosin positive deposits in the sub-RPE space of 24 m.o. (Figure 1H) and 5 m.o. CXCR5^−/−^ (Figure 1I) mice. These changes were apparent in the periodic acid Schiff (PAS)-stained sections (Figures 1K,L), appearing as large glycoprotein deposits in the RPE layer of 24 m.o. CXCR5^−/−^ mice (Figure 1K). In the 5 m.o. CXCR5^−/−^ mice; the deposits were more similar in shape and relative size to those of human AMD drusen (Figure 1L). In the age-matched WT control mice, H&E (Figure 1J) and PAS staining (Figure 1M) showed no abnormalities. Transmission electron microscopy (TEM) analysis of the RPE and retina (Figure 1N) from CXCR5^−/−^ (24 m.o.) mice revealed the presence of basal linear deposits. RPE cells showed marked vacuolization and an increased presence of microparticle objects. Furthermore, lymphocytes were observed within the choroid near Bruch's membrane. Ultrastructural disruption of photoreceptors (Figure 1O) was observed and was consistent with our previous TEM findings (22). In addition, photoreceptor fragments were noted within the intracellular space. Drusen-like deposits were observed in sub-RPE space (Figure 1P) and were similar in morphology and composition to drusen found in human AMD (23). A chain of vesicular structures, some consistent in structure with photoreceptor debris, appeared to originate in RPE cells and move toward drusen-like deposits; in some cases, vesicles appeared to enter the deposits.

### Retinal Flat Mount Findings

**Figure 2:**
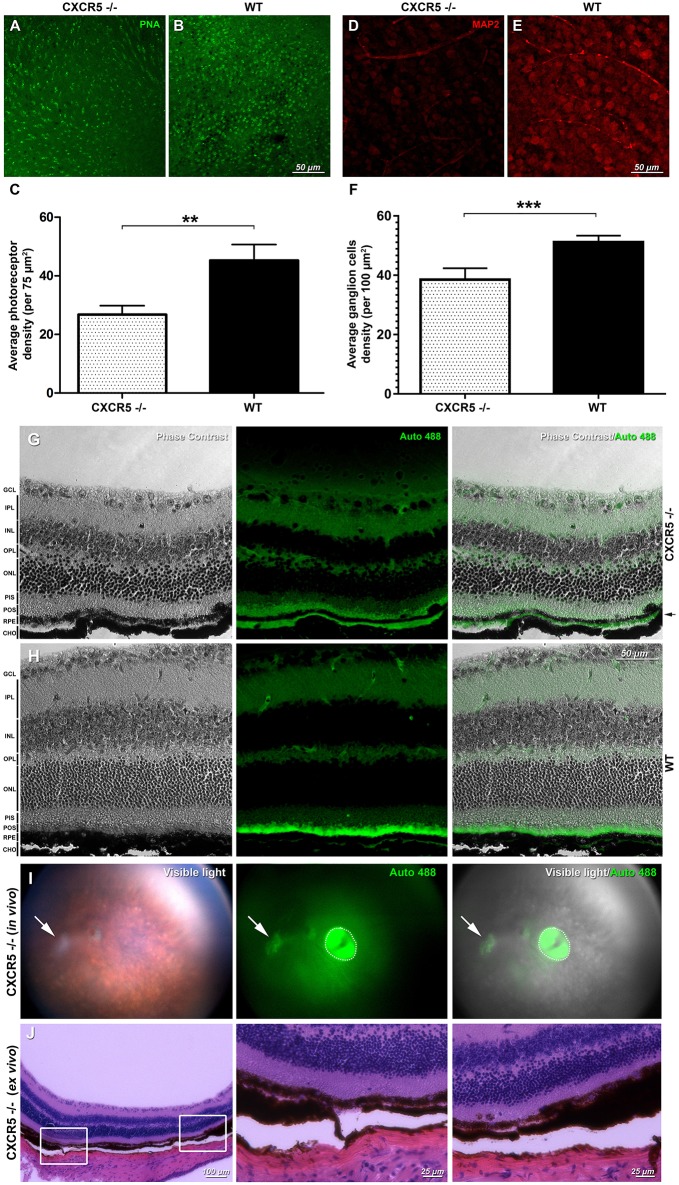
Retinal flat mounts evaluation and detection of autofluorescence and signs of geographic atrophy (GA) in aged CXCR5^−/−^ animals. Peanut lectin staining (PNA) of **(A)** CXCR5^−/−^ retinas demonstrated markedly reduced numbers of photoreceptors relative to the **(B)** WT retinas; PNA staining results are further confirmed by **(C)** quantitative analysis (*n* = 5) of the photoreceptors cells density; **(D)** MAP2 microtubule-associated protein 2 (MAP-2) immunostaining of CXCR5^−/−^ ganglion cells indicating marked reduction of the MAP-2 fluorescent signaling and number of ganglion cells when compared with the **(E)** WT controls; and consistent with **(F)** quantification of ganglion cells number (*n* = 5) that indicate significant reduction relative to the WT retinas. A student's *t*-test was used to determine statistical significance. **p < 0.01; ***p < 0.001. Phase contrast and autofluorescence images of the **(G)** CXCR5^−/−^ and **(H)** WT controls eye sections. Autofluorescent signal was detected in the sub-RPE space of 24 m.o. CXCR5^−/−^ mice and was visible as a second green layer separated by a black RPE layer from autofluorescent photoreceptors (indicated by black arrow). A marked reduction in the autofluorescence of the photoreceptor layer was also observed in aged CXCR5^−/−^ eyes. The minimal sub-RPE autofluorescent deposition was observed in age-matched WT controls. Retinal layer designations: GCL, ganglion cell layer; IPL, inner plexiform layer; INL, inner nuclear layer; OPL, outer plexiform layer; ONL, outer nuclear layer; PIS, photoreceptors inner segments; POS, photoreceptors outer segments; RPE, retinal pigment epithelia; CHO, choroid. All the samples were obtained from 24 m.o. female mice. Detection of autofluorescence in the eyes of 24 m.o. CXCR5^−/−^ mouse **(I)** the white arrow indicates the area of the atrophy, and the dotted circle indicates a reflection artifact. **(J)** Histological findings in the atrophied area of the same CXCR5^−/−^ animal demonstrating the RPE thinning and reduced density of the ganglion cells. Enlarged portions of the image demonstrate the beginning the end of the atrophic spot.

Peanut lectin staining (PNA) of CXCR5^−/−^ and WT retinas demonstrated loss of photoreceptor cells in CXCR5^−/−^ mice (Figure 2A) compared with WT mice (Figure 2B) (*p* < 0.01), per 75 μm^2^ in the peripheral retina (Figure 2C). A marked decrease (*p* < 0.01) in Microtubule-Associated Protein 2 (MAP2) signal and the number of MAP2 positive ganglion cells was observed in aged CXCR5^−/−^ mice (Figure 2D) compared with the WT age-matched control mice (Figure 2E); the corresponding quantification is shown in Figure 2F.

### Signs of Autofluorescent Sub-RPE Deposits and GA in CXCR5^−/−^ Mice

In the sub-RPE space of 24 m.o. CXCR5^−/−^ animals, autofluorescent signals were detected at 488 nm excitation wavelengths (Figure 2G) and were visible as the second green layer separated by the black RPE layer from the autofluorescent photoreceptors. A marked reduction in autofluorescence of the photoreceptor layer was also observed in aged (24 m.o.) CXCR5^−/−^ mice when compared with WT control mice, which are consistent with the photoreceptor loss indicated by PNA staining. The minimal sub-RPE autofluorescent deposition was observed in age-matched WT control mice (Figure 2H). A total of two out of 10 aged CXCR5^−/−^ female mice indicated abnormalities consistent with the geographic atrophy (GA) when examined by fundus images with presence of a large white spot in visible light, with autofluorescence at 488 nm excitation (Figure 2I) and H&E staining of the same eye (Figure 2J). The H&E staining also demonstrated RPE thinning and reduced ganglion cell density at the spot area.

### Immunofluorescent Analysis of AMD-associated Proteins

Eye section staining for C3d (Supplementary Figure 1A), Cryab (Supplementary Figure 1B), and Aβ (Supplementary Figure 1C) indicated an increased presence of these proteins within the sub-RPE space of 5 and 25 m.o. mice. The staining intensity appeared to increase with age, with the strongest signals observed in the sub-RPE space of 24 m.o. CXCR5^−/−^mice. Aged WT controls produced the noise levels of the signals for the investigated proteins.

### Immunofluorescent Analysis of RPE-choroidal-scleral Complex Flat Mounts

**Figure 3:**
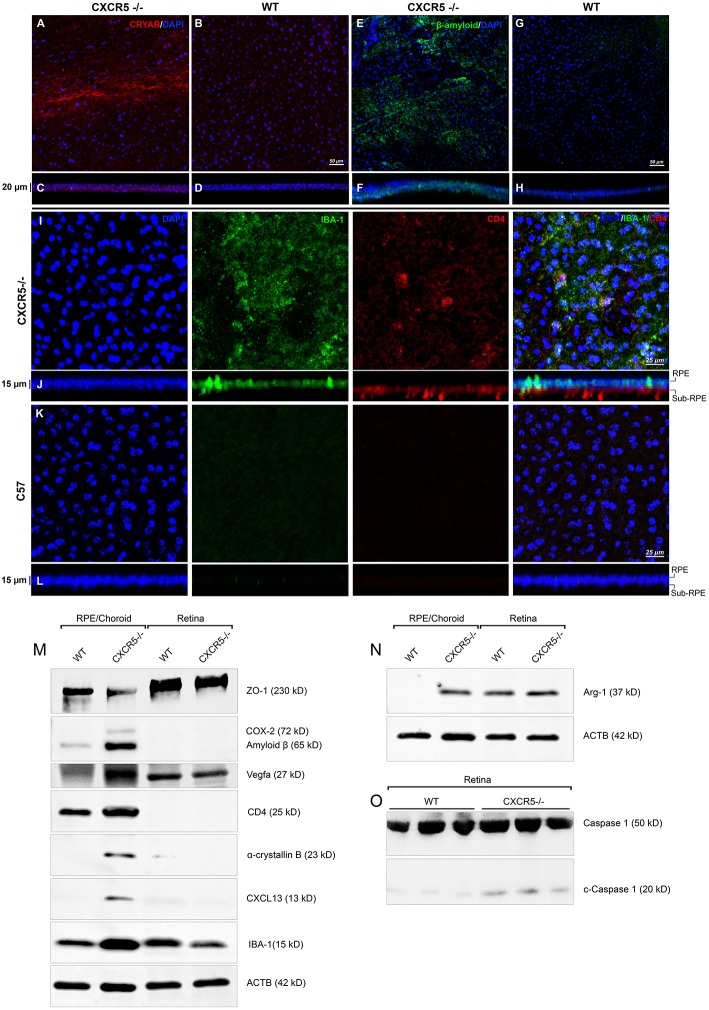
Immunofluorescent analysis of RPE/choroidal flat mounts and western blot analysis of RPE/Choroid and retinal material. Alpha-crystallin B (red) immunofluorescent staining in RPE/choroidal complexes. **(A)** Strong Alpha-crystallin B signals were detected in CXCR5^−/−^ animals; **(B,D)** noise levels of red fluorescence were detected in WT controls; z-stack depth analysis in **(C)** CXCR5^−/−^ flat mounts indicate sub-RPE localization of the signals in CXCR5^−/−^ RPE/choroidal complexes, forming a distinctive layer under the RPE cells. Marked amyloid beta (Aβ) staining (green) was detected in RPE/choroidal complexes in the **(E)** CXCR5^−/−^ sub-RPE localization of Aβ was identified by **(F)** z-stack depth analysis, no amyloid beta expression detected in **(G)** WT animals and **(H)** it's z-stacks. CXCR5^−/−^ mice also indicated an increased presence of IBA-1 (green) and CD4 (red) positive cells within the **(I)** RPE/choroidal complex; **(J)** z-stack analysis revealed the presence of IBA-1 positive signals on the surface of the RPE layer and sub-RPE localization of CD4 positive cells; **(K,L)** WT samples demonstrated minimal IBA-1 fluorescence and no CD4 positive cells. Nuclear counterstaining by DAPI (blue) in fluorescent images. Protein expression of **(M)** ZO-1, COX-2, CD4, Cryab, CXCL13, and VEGF-a; **(N)** Arg-1, expression CXCR5^−/−^ and age-matched WT control RPE/choroid and retina lysate. β-Actin used as a loading control. **(O)** Cleavage of Caspase-1 in retina lysate of CXCR5^−/−^ and age-matched WT control animals. All samples were obtained from 24 m.o. female mice.

Confocal microscopy of the RPE-choroidal-scleral complex (RCSC) flat mounts revealed the presence of Cryab (Figure 3A) and Aβ (Figure 3E) deposits in aged CXCR5^−/−^ mice. A z-stack analysis of the samples showed sub-RPE localization of the signal characteristic of a layer under the RPE cell nuclei (Figures 3C,F). No Aβ or Cryab signals were detected in the age-matched WT controls RCSC and its z-stacks (Figures 3B,D,G,H).

Using Griffonia simplicifolia (GSA)-lectin staining, an increase in the infiltration of GSA-lectin-positive cells was noted in aged CXCR5^−/−^ mice (Supplementary Figure 2A). This infiltration was not present in the WT controls (Supplementary Figure 2B). To identify the type and localization of these cells, IBA-1, and CD4 double immunostaining was performed. IBA-1 positive signals were observed on the surface of the RPE in CXCR5^−/−^ mice (Figure 3I). In addition, CD4 positive cells (CD4^+^) were detected in the sub-RPE layer by z-stack analysis (Figure 3J). This IBA-1 and CD4^+^ cellular infiltration were not present in the age-matched WT control RCSC (Figures 3K,L). The results were further confirmed in histological sections double-stained with IBA-1 and GSA-lectin of CXCR5^−/−^ WT control mice (Supplementary Figure 3A), where multiple, double-positive cells were detected in the subretinal space with no such infiltration present in the WT control sections (Supplementary Figure 2B).

### Western Blot Analysis of RPE/Choroid and Retinal Material

Western blot analysis indicated the presence of Aβ and Cryab in the RCSC of CXCR5^−/−^ mice, but these were absent in the RCSC lysate of age-matched WT controls (Figure 3M). Consistent with the confocal observations (Figure 3I), increased signals of IBA-1 and CD-4 were in the RCSC of CXCR5^−/−^ mice, indicating an increase of microglia and CD4-positive cells within the RCSC (Figure 3M). These data were further supported by an increased expression of the proinflammatory marker COX-2 (Figure 3M) and microglia activation marker Arg-1 (Figure 3N) in the RCSC, with a slight increase in expression of Arg-1 in the retina of CXCR5^−/−^ mice. An increase in VEGF-A signaling was detected in the RCSC of CXCR5^−/−^ mice (Figure 3M), which was consistent with histological observations shown in Figures 1H,I and TEM data (Figure 1N). Furthermore, increased expression of CXCL13 was found in RCSC (Figure 3M) but not in WT controls. The loss of blood-retinal barrier function (BRB) and RD were illustrated by a decrease in ZO-1 (Figure 3M) expression in the RCSC. In addition, no decrease in ZO-1expression occurred in the retina of aged CXCR5^−/−^ mice (Figure 3M); this is consistent with the *in vivo* fluorescence angiography results shown in Figures 1D,F. Increased cleavage of Caspase-1 was detected in retinal material from aged CXCR5^−/−^ mice (Figure 3O), indicative of pyroptosis.

### Endogenous Immunoglobulin G Detection and Antigen Protein Identification

**Figure 4:**
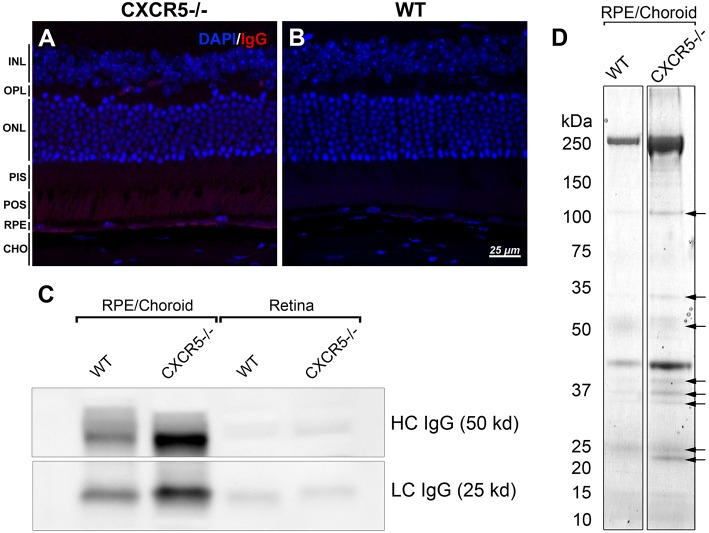
Detection of endogenous IgG and antigen identification. **(A)** Fluorescent staining detected the presence of endogenous IgG of CXCR5^−/−^; with no signals detected in **(B)** WT control sections. **(C)** Increased heavy chain (HC) and light chain (LC) of IgG accumulation in RPE/choroidal complex of 24 m.o. CXCR5^−/−^ mice when compared with age-matched WT controls. Sligh increase of HC IgG in the retina of CXCR5^−/−^ mice. **(D)** Protein blots were incubated with purified serum from 17 m.o. WT and CXCR5^−/−^ animals instead of a primary antibody for antigen identification. The identified bands (indicated by black arrows) were cut from the identical gel stained by Coomassie Blue and subjected to mass spectrometry analysis.

Previously, we reported endogenous Immunoglobulin G (IgG) detection in aged CXCR5^−/−^ mice with antimouse IgG secondary antibodies in protein blots not incubated with any primary antibody (22). Similarly, the histological sections of CXCR5^−/−^ eyes demonstrated increased fluorescence in the RPE/sub-RPE area (Figure 4A), but no such fluorescence was observed in WT age-matched controls (Figure 4B). These results were further supported by western blot analysis (Figure 4C). Consistent with our previous observations (22), the increased presence of the heavy chain (HC) and light chain (LC) IgG were detected in the RCSC lysate of aged CXCR5^−/−^ mice. To identify the protein composition in the RCSC that endogenous antibodies are produced to we performed a modified western blot process in which protein blots were incubated with purified serum (instead of primary antibodies) from WT and CXCR5^−/−^ aged mice to any specific protein. The resulting visualization of the observed protein bands is presented in Figure 4D. The distinctive bands identified within the membrane by chemiluminescence were cut from the twin gel with the same sample stained with Coomassie Brilliant Blue (Coomassie) and subjected to mass spectrometry analysis to identify the antigen composition. Eleven of the RPE antigens that were identified by mass spectrometry within the bands of CXCR5^−/−^ RCSC sample are presented in Table 1.

**Table 1:** Eleven RPE antigens identified with autologous serum precipitation and mass spectrometry analyses in aged CXCR5^−/−^ choroid-scleral complexes.

**Uniprot ID#**	**Gene symbol**	**Protein name**	**Drusen component**	**AMD association**	**Description**
P23927	Cryab	Alpha-crystallin B	+	+	Molecular chaperone (24, 25)
P24622	Cryaa	Alpha-crystallin A	+	+	Molecular chaperone (24, 25)
P07356	Anxa2	Annexin A2	+	+	Regulation of phagocytosis (26)
POCG49	Ubb	Ubiquitin-B	+	+	Protein degradation (27)
Q61495	Dsg1a	Desmoglein 1 aplha	?	?	Desmosome component (28, 29)
E9Q557	Dsp	Desmoplakin	?	?	Desmosome component (28, 29)
Q02257	Jup	Junction plakoglobin	?	?	Junction plaque protein (30)
Q9WTY4	Aqp5	Aquaporin-5	?	?	Water-specific channel (31)
Q60847	Col12a1	Collagen apha-1 (XII) chain	?	?	Extracellular matrix (32)
Q922J3	Clip1	CAP-Gly linker protein 1	?	?	Regulation of endosome trafficking (33)
Q9ESN9	Mapk8ip3	JNK-interacting protein	?	?	Regulation of vesicle transport (34)

*Autologous serum precipitation and mass spectrometry analysis identified Alpha-crystallin B, Alpha-crystallin A, Annexin A2, and Ubiquitin-B, which are known AMD drusen components as antigens to circulating autoantibodies in 24 m.o. CXCR5^−/−^ animals. Remaining 7 proteins are not implicated in AMD pathology, but related to the regulation of extracellular matrix, barrier function, and water balance*.

### *In vitro* Response of BV-2 Cells to Activation When Treated With CXCR5 Antibodies

Finally, we investigated whether microglia cells respond differently to stimulation under antibody-mediated inhibition of CXCR5 in BV-2 cells line. BV-2 cells can express Arginase-1 (Arg-1) and Cyclooxygenase-2 (COX-2) when stimulated with recombinant mouse interleukin-4 (rmIL-4), and recombinant mouse TNF-α (rmTNF-α) supplemented with recombinant mouse interferon gamma (rmIFNγ) (35–37). Treatment with the CXCR5 antibody alone did not result in a noticeable increase of Arg-1 or COX-2 (Supplementary Figures 4A,D). As expected, rmIL-4 and rmTNF-α/rmIFNγ treatments upregulated the expression of Arg-1 and COX-2, respectively (Supplementary Figures 4B,E). However, a stronger expression of Arg-1 and COX-2 was observed in CXCR5-antibody-treated cultures than in the positive control (Supplementary Figures 4C,F), and this was further confirmed with RT-PCR (Supplementary Figures 4G,H), Arg-1 (*p* < 0.001), and COX-2 (*p* < 0.01).

## Discussion

In this study, we characterized RD phenotype in aged CXCR5^−/−^ mice, which included retinal and RPE cell degeneration, drusen-like deposits, and—most crucially—the presence of Aβ and Cryab in sub-RPE space. Aβ is of particular importance because it is reported to cause activation of the alternative complement cascade (38), and it is shown to be expressed in human AMD pathophysiology; therefore, Aβ is a potential target for therapeutic intervention (39–41). For complement activation, we identified complement component 3 (C3d) in the sub-RPE space of aged CXCR5^−/−^ mice. C3d plays a critical role in enhancing B cell-specific immune responses (42). We also detected Cryab in the sub-RPE space and within the RCSC of the aged CXCR5^−/−^ mice. One of the AMD-associated proteins, Cryab, is a target for microglia adaptive immune responses through the stimulation of secretion leukocyte-recruiting factors (43).

WB analysis of the RCSC in aged CXCR5–/– mice identified increased expression of CXCL13. CXCL13 is a ligand of CXCR5 and is found in B cell aggregates that develop in the inflamed meninges of mice with experimental autoimmune encephalomyelitis (EAE) (44) and in humans with progressive multiple sclerosis (MS) (45). The autoimmune reaction in chronic uveitis attributed to the CXCR5-CXCL13 system is also reported in the mouse retina with CD4^+^ cell infiltration (46). Experiments with the forced expression of CXCL13 in non-lymphoid organs led to the recruitment of immune cells into the affected tissue (47). Consistently, our WB analysis showed increased expression of both IBA-1 microglia markers and CD4^+^ signaling within the RCSC of CXCR5^−/−^ mice. Localization with immunofluorescent staining showed IBA-1 positive microglia cells on the surface of RPE and CD4^+^ cells in the sub-RPE space.

The increased expression of Arg-1 and COX-2 in RCSC of aged CXCR5^−/−^ mice is suggestive of increased microglia activation. Our *in vitro* experiments in BV-2 cells indicate that microglia, when treated with the CXCR5 antibody, have a stronger response to stimuli such as IL-4 and TNFα/IFNγ in the production of Arg-1 and COX-2, respectively. However, when cultures were treated with CXCR5 antibody without stimulating agents, no notable response in microglia activation was observed; this suggests that CXCR5 may have a regulatory role in microglia cells proinflammatory activation. Hypersensitivity of microglia cells to stimuli, along with the overproduction of CXCL13, may be a factor leading to the chronic damage of photoreceptors and RPE cells, causing the migration of microglia toward the photoreceptor and RPE layers of the eye. Interestingly, our previous study identified microglia cells positive for F4/80 on the surface of the RPE, which is colocalized with RPE65; at the time, we could not definitively identify the cause of this double signaling (22). In mice, RPE65 is usually present in RPE cells, as well as in the photoreceptors (48). Currently, we believe that this is an indication of microglia phagocytosis of the photoreceptor and RPE cell material. Autophagy and associated cell death are implicated in AMD pathology (49). Increased cleavage of Caspase-1 in the retina of aged CXCR5^−/−^ mice is indicative of immune cells mediated pyroptosis. Ardeljan et al. (50) suggest that pyroptosis is the predominant mechanism of cellular death in AMD along with necroptosis. Dying photoreceptors and other cell types debris are absorbed by microglia, causing it further activation and RPE cells and eventually deposited in sub-RPE space.

Initially, the inflammatory process within the retina and RPE remains locally isolated by BRB; however, constant subacute proinflammatory signaling compromises the integrity of the BRB tight junctions, characterized by downregulation of junctional proteins such as ZO-1. This exposes Cryab, Cryaa, and other antigens to the adaptive immune system leading to the formation of autoantibodies that result in a specific autoimmune reaction in the aged CXCR5^−/−^ animals. Our previous and current findings indicate that the marked reduction of ZO-1 in the RCSC of aged CXCR5^−/−^ mice, increased terminal deoxynucleotidyl transferase dUTP nick end labeling (TUNEL) signals in the retina, and a decrease of a-wave amplitude in electroretinography (ERG) progresses with age (22).

**Figure 5:**
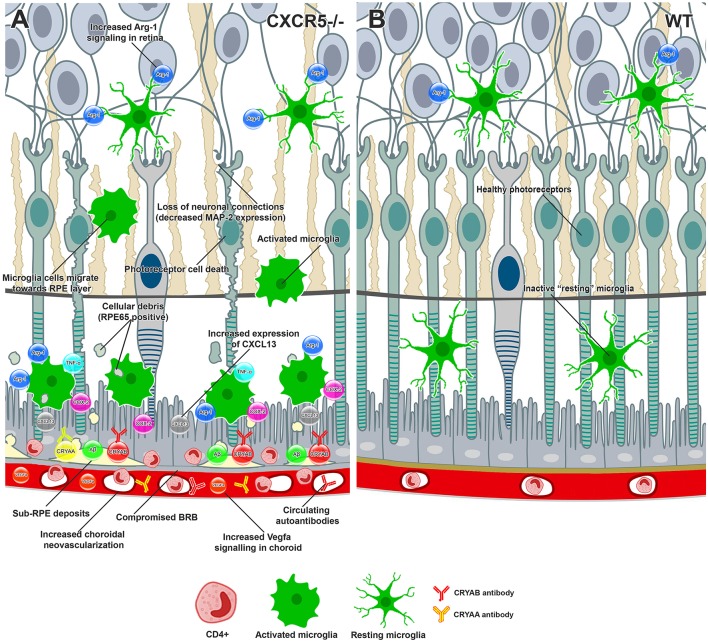
Conceptual summary of the proposed cellular events in aged CXCR5–/– retinas and choroid based on the findings of the current study. **(A)** CXCR5^−/−^ microglia cells demonstrated upregulation of Arg-1 in the retina. Increased production of CXCL13-activated microglia cells migrating toward the RPE, releasing COX-2, TNFa, and excessive amounts of Arg-1. RPE and photoreceptor cells were affected by cytokine signaling, triggering cell death. The loss of photoreceptors and subsequent neuronal connections led to reduced MAP-2 signaling within the retina. RPE65 positive cellular debris of dying photoreceptors subjected to phagocytosis by microglia cells and eventually deposited in the sub-RPE space, triggering the accumulation of amyloid beta, Cryaa, Cryab, and other AMD-associated proteins. Constant proinflammatory signaling within the RPE layer affected the tight junction proteins, including ZO-1, compromising the BRB and making drusen components accessible to the immune system and production of specific autoantibodies. The binding of autoantibodies to the components of sub-RPE deposits triggered infiltration with CD4-positive inflammatory cells. Constant VEGF signaling within the choroid led to the release of VEGF-a and choroidal neovascularization. **(B)** Healthy retina and RPE layers of WT animal.

We previously demonstrated that increased levels of heavy and light chains of IgG are the first indication an autoantibody in 17 m.o. RCSC CXCR5^−/−^ mice (22); our current work confirms this. Here, we noted the presence of systemic circulating autoantibodies to Cryaa, Cryab, Ubiquitin-B, and Annexin 2 in the serum of aged CXCR5^−/−^ mice; these findings are consistent with those in recent studies by Iannaccone et al. (51) in human AMD patients and with other studies demonstrating the upregulation of Cryaa and Cryab in Bruch's membrane and the choroidal tissues of AMD patients (24, 25). The process becomes a self-sustained inflammatory loop in which more proinflammatory events in the retina and RPE lead to increased activation of microglia, deposition of the cellular debris, further impairment of BRB, and increased autoantibody formation. A graphical summary of the findings and proposed molecular events are presented in Figure 5.

Limitations of this study: (1) We demonstrated findings suggestive of microglia activation and migration toward the RCSC in the aged CXCR5^−/−^ mice; however, multiple time points at different ages are required to fully elucidate the role and dynamics of microglia and CD4^+^ cell populations activation and migration in the CXCR5^−/−^ RD process. (2) *in vitro* results, while supportive of *in vivo* findings, require further confirmation in primary retinal microglia cells obtained and cultured from CXCR5^−/−^ and WT mice. This is due to the potential difference in the functional response of brain-derived BV-2 cell line and retinal microglia cells.

## Conclusion

Together, our findings suggest that aged CXCR5^−/−^ mice may be a useful animal model for studying the autoimmune aspects of RD in mice. This animal model has an AMD-like retinal pathology that demonstrates complementary system activation, robust drusen-like aggregates in the sub-RPE space consistent in structure with human pathology, and accumulation of AMD-associated proteins such as Aβ and Cryab. This animal model also has autoimmune-driven degenerative changes with systemic circulating autoantibodies to Cryaa, Cryab, Annexin A2, and others.

## Materials and Methods

### Animals

The [B6.129S2(Cg)-Cxcr5tm1^Lipp/J^] (CXCR5^−/−^) and [C57BL/6J] (WT) mice strains were purchased from Jackson Laboratory. B6.129S2(Cg)-Cxcr5tm1^Lipp/J^ mice are on a C57/BL6J background with a small component of C57/BL6N genes (http://jaxmice.jax.org/strain/006659.html). The CXCR5 gene was replaced by the neomycin resistance gene in 129S2/SvPas-derived D3 embryonic stem cells. Resulting mutant mice were then backcrossed to C57BL/6 mice for 8 generations (52). Both CXCR5^−/−^ and WT mice were housed at the special pathogen-free Cancer Research Building Animal Facilities at Johns Hopkins Hospital. All mice were fed normal chow diets and provided with water *ad libitum*. During experimentation, the mice were anesthetized with ketamine hydrochloride (100 mg/kg body weight) and xylazine (4 mg/kg body weight). All experiments were approved by the Institutional Animal Care and Use Committee of Johns Hopkins University School of Medicine (protocol number: M016M480) and were in accordance with the “Statement for the Use of Animals in Ophthalmic and Vision Research” of the Association for Research in Vision and Ophthalmology.

### Genomic DNA Extraction and Genotyping

Genomic DNA (gDNA) was extracted from tail tip material (approximately 1–2 mm in length) from WT and CXCR5^−/−^ animals using the DNEasy blood and tissue extraction kit (Qiagen, Gaithersburg, MD, USA) according to the manufacturer's instructions. Extracted DNA was quantified and evaluated for purity using a NanoDrop One spectrophotometer (Thermo Fisher Scientific, MA, USA) and analyzed as reported previously (22). Further validation was performed with the assistance of Transnetyx: Outsourced PCR Genotyping Services (www.transnetyx.com) by real-time PCR genotypic assay. Genomic DNA (50 ng) was amplified using TaqMan™custom-designed genotyping primers (TransnetYX) complementary to the sequence fragments of CXCR5 and neomycin resistance gene. The probe concentration in the final PCR well was 250 nM. The RT-PCR reaction condition was as follows: reaction activation 50°C for 2 min, initial DNA denaturation 95°C for 10 min (95°C 15 s, 60°C 1 min), and repeat for 40 cycles using a TaqMan™ Fast master mix. CXCR5^−/−^ and WT animals were validated for knockout of the CXCR5 gene and the presence of the neomycin resistance gene, along with the absence of neomycin resistance and the presence of CXCR5 in the WT controls, respectively.

### DNA Sanger Sequencing for Detection of rd8-associated Nucleotide Deletion

The genomic DNA template (30 ng) was amplified using PCR. The reaction mix was as follows: 12.5 μl of PfuUltra II Hotstart PCR Master Mix (Agilent), 1.2 μM of each oligo (Crb1-3511: 5′ CCCTGGTAAGCCTCAGGAAG 3′ and Crb1-mR: 5′ GCCCCATTTGCACACTGATGAC 3′), and DNase/RNase free ultra-pure distilled water (Invitrogen) to equilibrate the reaction volume to 25 μl. PCR amplification was performed using Applied Biosystems SimpliAmp Thermal Cycler (Thermo Fisher Scientific). The resulting products were validated for correct amplification by running 3 μl of the amplified product on 4% agarose E-Gel EX SYBR Gold II (Thermo Fisher Scientific) along with a TrackIt™1 Kb Plus DNA Ladder (Thermo Fisher Scientific) using the E-Gel Power Snap Electrophoresis System (Thermo Fisher Scientific). Successful amplification was determined by the presence of a single sharp and bright band of approximately 300 bp. The products and Crb1-3511 primer at 0.4 μM concentration were sent to the Synthesis & Sequencing Facility (Johns Hopkins University) for purification with an AMPure XP magnetic beads system and Sanger sequencing. The resulting data were aligned and cross-referenced with wild-type sequence NM_133239 of the Crb1 gene using SnapGene software (http://www.snapgene.com). The absence of an RD8 mutation in the CRB1 gene was verified by the identification of cytosine at position 3647. A representative fragment of Sanger sequence data (Supplementary Figure 4A) of CXCR5^−/−^ animal was aligned with the fragment of the canonical sequence of the CRB1 gene (NM_133239) (Supplementary Figure 4B); missing “C” at position 3647 and one letter frameshift were indicative of the CRB1-RD8 mutants. The presence of double peaks at and after the mutation site were recognized as heterozygotes.

### Fundus Examination and Florescent Angiography With the Retinal-Imaging Microscope

Mice were anesthetized intraperitoneally (i.p.) with Ketanest (ketamine; 25 mg/ml, 0.4 ml, Pfizer, NY, USA) and Dexdomitor (dexmedetomidine hydrochloride; 0.5 mg/ml, 0.2 ml, Orion Pharma, Hamburg, Germany). Pupils were dilated with 1% tropicamide (Sandoz, US). The cornea was protected with (hypromellose ophthalmic demulcent solution) Gonak 2.5% (Akorn LLC, Akorn, OH, USA) transparent gonioscopy gel. Fundus examination was performed with a Micron III retinal-imaging microscope (Phoenix Research Labs, Inc., Pleasanton, CA, USA). Drusen-like deposits were later counted in the resulting digital images. Following the acquisition of visible light images, mice were injected subcutaneously with 100 μl of 5% sodium fluorescein (Alcon Laboratories, Fort Worth, TX) per animal. Fundus vascular fluorescence was observed using 488 nm excitation with a 520 nm emission filter. The observation of fundus autofluorescence was performed as described above without the injection of sodium fluorescein.

### Imaging

Visible light images were acquired using Zeiss AxioPhot (Carl Zeiss, Oberkochen, Germany) and EVOS FL Color microscopes (Thermo Fisher Scientific). Fluorescent images and z-stacks were acquired with an LSM 700 laser confocal microscope (Carl Zeiss, Oberkochen, Germany).

### Histology and Immunofluorescent Analysis

Anesthetized mice were euthanized by subjecting them to a carbon dioxide (CO_2_) atmosphere. The harvested eyeballs were fixed in 4% formaldehyde (Sigma-Aldrich, St. Louis, MO, USA) overnight at 4°C and stored in PBS until processed for paraffin embedding and sectioning (5 μm thick). Sections were stained with H&E. Sections intended for PAS staining were rehydrated, treated with periodic acid for 5 min, washed with water, covered with Schiff's reagent for 8 min, dehydrated, and mounted with quick dry mounting media (Thermo Fisher Scientific).

Sections intended for immunohistochemical analysis were rehydrated; heat-induced antigen retrieval was performed in a 1x citrate buffer (pH 6.0) (Citrate Buffer [pH 6.0], Concentrate 10x; 005000; Thermo Fisher Scientific). The sections were permeabilized by incubating with 0.2% Triton-X100 (Sigma-Aldrich) in PBS for 15 min, incubated for 30 min with Image-iT™FX Signal Enhancer (Thermo Fisher Scientific) at RT, then blocked with 5% normal goat serum (NGS) (Thermo Fisher Scientific) for 1 h at RT. Samples were incubated with beta-amyloid (36-6900; 1:100, Thermo Fisher Scientific), crystallin alpha B (Ab151722, 1:50; Abcam), and C3d (AF2655, 1:100, R&D) antibodies and then were visualized by Cy5 conjugated secondary antirabbit (ab97077) and antimouse (ab6563) secondary antibody 1:1,000 (Abcam). Sections were counterstained with 4′, 6-diamidino-2-phenylindole (DAPI) 1:5,000 (Sigma-Aldrich) and mounted with ProLong Diamond antifade reagent (Invitrogen, Thermo Fisher Scientific).

### Retinal Flat Mounts

Fixed eyeballs were dissected to isolate the retina from the eyecup. The retina was permeabilized within a blocking solution composed of 5% NGS in PBS overnight with 0.01% Triton-X; the samples were removed from a blocking solution and washed with PBS and then incubated with FITS-conjugated peanut lectin (Vectorlabs, USA) for 2 h at RT. Following washing with Tween 20 0.02% in PBS (PBS-T), the retinas were mounted photoreceptor side up. After the blocking, the retinas intended for MAP2 immunostaining were incubated with the anti-MAP2 (M13, 13-1500; 1:100; Abcam) antibody for 24 h, followed by washing with PBS-T three times for 10 min and then incubation for 24 h with (ab6563) secondary antibody 1:1,000 in 2.5% NGS (Thermo Fisher Scientific) and DAPI 1:5,000 (Sigma-Aldrich). After another PBS-T washing, the samples were mounted with ProLong Diamond antifade reagent (Invitrogen) on slides with the photoreceptor side down.

### RCSC Flat Mounts

The eyeballs were fixed with 4% paraformaldehyde (Sigma-Aldrich) for 12 h. Under an Olympus SZ-STB1 (Olympus) dissection microscope, the anterior segment tissues, vitreous, and retinas were removed to isolate the RCSC. Approximately four to eight relaxing radial incisions were made, and the remaining RCSC were incubated overnight in a blocking solution composed of 5% NGS (Thermo Fisher Scientific) with 0.01% Triton-X (Sigma-Aldrich). The RPE-choroidal-scleral complexes were then incubated with beta-amyloid (36-6900; 1:100; Thermo Fisher Scientific), crystallin alpha B (Ab151722, 1:50; Abcam), Iba1 (019-19741; 1:100; Wako), and Anti-CD4 (MA5-12259, 1:100, Thermo Fisher Scientific). The RPE-choroidal-scleral complexes were then incubated with primary antibody dissolved in 2.5% NGS (Thermo Fisher Scientific) for 24 h before being washed three times for 10 min with PBS-T; then, the samples were incubated for 24 h with Cy5 conjugated secondary antirabbit (ab97077) and antimouse (ab6563) secondary antibody 1:1,000 in 2.5% NGS (Thermo Fisher Scientific) and DAPI 1:5,000 (Sigma-Aldrich). After another PBS-T washing, three times for 10 min, the samples were mounted on slides with ProLong Diamond antifade reagent (Invitrogen).

### Transmission Electron Microscopy

The eyes were enucleated and fixed with 2.5% glutaraldehyde and 2.5% paraformaldehyde (in 0.1 M cacodylate buffer, pH 7.4) for TEM. The anterior segment and vitreous humor were removed. The eyecups with the retina, RPE, and choroid were fixed with 1% osmium tetroxide in a 0.1 M cacodylate buffer (pH 7.4). The fixed eyecups were dehydrated with gradient alcohols and embedded in Poly/Bed 812 resin. Next, 70-nanometer ultrathin sections were cut with a Leica EM UC 7 microtome and stained with uranyl acetate and lead citrate. The stained specimens were analyzed with a Hitachi H-7600 TEM instrument (Hitachi Co. Ltd., Tokyo, Japan).

### Western Blot Analysis

Immediately following euthanasia of the mice, the eyes were enucleated and dissected on ice, carefully isolating the retina and RPE/choroid complex. The samples were lysed immediately or stored at −80°C until use. Lysis was performed in a RIPA buffer (Thermo Fisher Scientific) supplemented with a 1:100 Protease inhibitor cocktail (Cell Signaling Technology); disruption of the tissue material was performed by a Q55 Sonicator (Qsonica, NY, USA) with four pulses for 22 kHz, 5 s each at 30% power output on ice in the 4°C environment (cold room). The resulting protein extracts were measured by Qubit® 4.0 Fluorometer (Thermo Fisher Scientific), and 20 μg of total protein were separated by SDS-PAGE (Mini-Protean Precast Acrylamide Gels, Bio-Rad, CA, USA). The gel-separated proteins then were transferred onto a nitrocellulose membrane (Trans-Blot Turbo transfer pack, Bio-Rad). The membrane was blocked with 5% skim milk (Bio-Rad) and probed with antibodies against COX-2 (MA5-14568; 1:1,000; Thermo Fisher Scientific); beta-amyloid (36-6900; 1:100, Thermo Fisher Scientific); VEGF-a (A-20, sc-152, 1:1,000, Santa Cruz Biotechnology); crystallin alpha B (Ab151722, 1:500; Abcam); anti-Iba1 (019-19741; 1:1,000; Wako); anti-CD4 (MA5-12259, 1:100, Thermo Fisher Scientific); CXCL13 (PA5-47018; 1:500; Thermo Fisher Scientific); Arginase 1 (24HCLC; 1:1,000; Thermo Fisher Scientific); Caspase-1 (2225, 1:1,000, Cell Signaling Technology); cleaved Caspase-1 (Asp297, D57A2, 1:1,000, Cell Signaling Technology); Rabbit mAb #4199 or β-actin (PA1-21167; 1:2,000; Thermo Fisher Scientific) at 4°C overnight. The target protein bands were detected with HRP-conjugated IgG antibody (170-6515, 172-1011, 1720-1011, 1:1,000; Bio-Rad), which was visualized by chemiluminescence with Clarity Western ECL substrate (Bio-Rad) and imaged using the LAS-500 Imaging System (General Electric, CT, USA). The resulting band sizes were resolved using PM2600 (Green BioResearch, USA) protein standard. Restore Western Blot Stripping Buffer (Thermo Fisher Scientific) was used between the incubations.

### Endogenous IgG Detection in Histological Sections and Tissue Lysate

Histological sections were rehydrated, and heat-induced antigen retrieval was performed in 1x citrate buffer (pH 6.0) (Citrate Buffer pH 6.0; 005000; Thermo Fisher Scientific). Sections were permeabilized by incubating with 0.2% Triton-X100 (Sigma-Aldrich) in PBS for 15 min and then blocked with 5% NGS (Thermo Fisher Scientific) for 1 h at RT. No primary antibodies were used. Samples were incubated overnight at 4°C with Cy5 conjugated antimouse (ab6563, 1:1,000, Abcam) secondary antibody. After washing in PBS-T, the samples were counterstained with DAPI 1:5,000 (Sigma-Aldrich) and mounted.

Western blot on RCSC and retinal lysates were performed as described above. After blocking, membranes were incubated with Goat Anti-Mouse IgG (Heavy + Light)-HRP conjugate (170-6516, 1:1,000; Bio-Rad) and detected using the LAS-500 Imaging System (General Electric).

### Antigen Identification by SDS-PAGE Separation and Mass Spectrometry Analysis

Blood samples (approximately 1 ml) from the 17 m.o. CXCR5^−/−^ mice and the age-matched WT mice were collected intracardially under terminal anesthesia. Ketanest (Pfizer) and Dexdomitor (Orion Pharma) were collected into the lithium-heparin tube (Terumo VF-054SHL) and were supplemented with 3 μL of protease cocktail inhibitor. The cellular components of the blood were removed by centrifugation at 3,000 rpm for 20 min at 4°C, resulting in mouse blood plasma samples that were collected and stored 3 h at 4°C until use. The eyes of the animals that the plasma samples were collected from were enucleated during the plasma preparation; the RPE/choroid complexes were dissected and extracted as described in section Western Blot Analysis. Total proteins (30 μg) were loaded onto two 10% SDS-PAGE (Mini-Protean Precast Acrylamide Gels, Bio-Rad) gels. Gel-separated proteins on one gel were transferred onto a nitrocellulose membrane (Trans-Blot Turbo transfer pack, Bio-Rad). The second gel was stored at 4°C until use the next day. The membrane was blocked with 3% BSA for 1 h; then, the membrane was cut, and the membrane strips with CXCR5^−/−^ and WT RPE/choroid were incubated overnight with 1:5 dilution (in PBS) of autologous plasma overnight. The next day, the samples were washed with PBS-T and incubated with the anti-mouse HRP-conjugated antibody (172-1011, 1:1,000; Bio-Rad) and visualized by chemiluminescence with Clarity Western ECL substrate (Bio-Rad). The duplicate gel was released from the cassette and stained with Coomassie Blue (Bio-Rad). The eight gel fractions that corresponded to the increased protein bands in the CXCR5^−/−^ immunoblotting membrane were cut and further processed for antigen identification by mass spectrometry.

### Cells and Cell Culture Conditions

Immortalized mouse microglia cell line BV-2 (EOC-20; CRL-2469; Lot: 70005904; ATCC), which is derived from the C3H/HeJ female mouse, was used at passage 5. BV-2 cell line DNA was verified to be absent from the CRB1-RD8 mutation by a custom-designed SNP RT-PCR probe (TransnetYX: Outsourced DNA Genotyping Services). BV-2 cells were maintained in culture DMEM (Gibco, Thermo Fisher Scientific) and supplemented with 10% FBS (Gibco, Thermo Fisher Scientific), 1% Pen/Strep (Gibco, Thermo Fisher Scientific), and 20% LADMAC conditioned media (LCM). Mouse bone marrow derived from the macrophage cell line LADMAC (CRL-2420; Lot: 63407846; ATCC) was used as a source of crude CSF-1 to supplement BV-2 cell growth. LADMAC cells were grown to confluency in complete DMEM; then, the media was changed to fresh complete DMEM, and 24 h later, conditioned media was collected and centrifugated at 5,000 G for 10 min to remove cells and debris, resulting in supernatants that were further filtered through a Target2™0.2 μm pore size and syringe-driven filter (F2513-2, Thermo Fisher Scientific). The resulting LCM was stored at −20°C until use.

### BV-2 Cells Treatment With CXCR5 Antibody and Stimulation With IL-4 and TNF-α and IFNγ *in vitro*

Here, 5 × 10^5^ BV-2 cells were seeded into six-well plates, and 5 × 10^3^ were seeded onto a Millicell EZ slide (Millipore) in LCM and grown to 80% confluency. Cells were treated with CXCR5 antibody low endotoxin, sodium azide-free (ab225575, 1:100, Abcam). Mouse IgG (ab37355, 1:100, Abcam) was used as a control. Following a 6-h incubation, the LCM medium was removed, and the cells were washed with PBS, and fresh FBS free DMEM media was supplied. Cell cultures were then stimulated with 10 ng/ml of recombinant mouse interleukin-4 rmIL-4 (PMC0045 Thermo Fisher Scientific), and the other wells were treated with 20 ng/ml of recombinant mouse TNF-α (rmTNF-α, PHC3016, Thermo Fisher Scientific) and supplemented with 10 ng/ml of recombinant mouse interferon gamma (rmIFNγ, PMC4034, PHC3016, Thermo Fisher Scientific). Following 12 h of incubation, the cells were washed with PBS, and the total RNA was extracted and purified (RNeasy Purification Kits; Qiagen) using RT-PCR analysis.

BV-2 cells that were seeded into Millicell EZ slides were washed with PBS and fixed with 2% formaldehyde (Sigma-Aldrich) for 10 min for immunocytochemistry analysis.

### Arg-1 and COX-2 Expression Profile in BV-2 Cells Following Recombinant Mouse IL-4 and TNF-α/INFγ Stimulation

RNA was analyzed for quality and quantified using a NanoDrop One (Thermo Fisher Scientific) and reverse transcripted to cDNA according to the manufacturer's protocol (SuperScript VILO cDNA Synthesis Kit; Invitrogen, CA, USA, in SimpliAmp Thermal Cycler; Life Technology, MA, USA). Gene expression analysis was performed using Power SYBR Green Master Mix (Thermo Fisher Scientific) with the following mouse-specific primers:

Arg-1 (forward: GGAATCTGCATGGGCAACCTGTGT, reverse: AGGGTCTACGTCTCGCAAGCCA), COX-2 (forward: GCGAGCTAAGAGCTTCAGGA, reverse: CAGACGCCACTGTCGCTTT), and Cyclophilin (forward: CAGACGCCACTGTCGCTTT, reverse: TGTCTTTGGAACTTTGTCTG) in a Quant Studio 3 RT-PCR system (Applied Biosystems, CA, USA). The relative expression values of target genes were normalized to cyclophilin, and the fold change was calculated using the relative quantification (2–ΔΔCT) method. Four biological replicates per treatment group were run in three technical replicates.

BV-2 cells that were seeded into Millicell EZ (Sigma-Aldrich) slides were fixed with 2% formaldehyde (Sigma-Aldrich) for 10 min, permeabilized by incubating with 0.2% Triton-X100 (Sigma) in PBS for 15 min, and then blocked with 5% NGS (Thermo Fisher Scientific) for 1 h at RT. The samples were incubated with COX-2 (MA5-14568; 1:1,000; Thermo Fisher Scientific) and Arg-1 (24HCLC, 1:100, Thermo Fisher Scientific) antibody overnight; then the antibodies were visualized by Cy5 conjugated secondary antirabbit (ab97077) secondary antibody 1:1,000 (Abcam). The sections were counterstained with DAPI 1:5,000 (Sigma-Aldrich) and mounted.

### Statistical Analysis

All values were expressed as the mean ± standard deviation (SD) for the respective groups. Statistical analyses were performed with GraphPad Prism software (https://www.graphpad.com/scientific-software/prism/). The Student's *t*-test was used whenever comparing two groups. A one-way ANOVA with Tukey multiple comparisons was used whenever comparing multiple groups. A *p*-value of <0.05 was considered significant. The following designations for the *p*-value were used in the manuscript figures: n.s. *p* > 0.05; ^*^p < 0.05; ^**^p < 0.01; ^***^p < 0.001.

## Ethics Statement

All experiments were approved by the Institutional Animal Care and Use Committee of Johns Hopkins University School of Medicine (protocol number: M016M480) and were in accordance with the guidelines of the Association for Research in Vision and Ophthalmology Statement for the use of animals in ophthalmic and vision research.

## Author Contributions

The study was conceived and designed by HH. AL and MS have conducted genotyping and sequencing analysis. HH and AL have conducted *in vivo* experiments. AL and AM performed histological staining, confocal experiments, and WB analysis. AL and MS performed *in vitro* experiments and associated analysis. HH performed endogenous IgG detection and Mass Spectrometry data analysis. The manuscript was written by AL, MS, AM, ST, and HH and critically revised by HH and ST. All authors reviewed and accepted the final version of the manuscript.

### Conflict of Interest Statement

The authors declare that the research was conducted in the absence of any commercial or financial relationships that could be construed as a potential conflict of interest.
