# Supplementary material for: Autoimmune-Mediated Retinopathy in CXCR5-Deficient Mice as the Result of Age-Related Macular Degeneration Associated Proteins Accumulation
Source: Front Immunol. 2019 Aug 14;10:1903. doi: 10.3389/fimmu.2019.01903 (PMC6702970; doi:10.3389/fimmu.2019.01903)
Supplement: Supplementary file 1 [file Data_Sheet_1.docx]

Supplementary Material

Autoimmune-mediated retinopathy in CXCR5-deficient mice as the result of accumulation of age-related macular degeneration associated proteins

Anton Lennikov, Madhu Sudhana Saddala, Anthony Mukwaya, Shibo Tang, Hu Huang

## Supplementary Figures


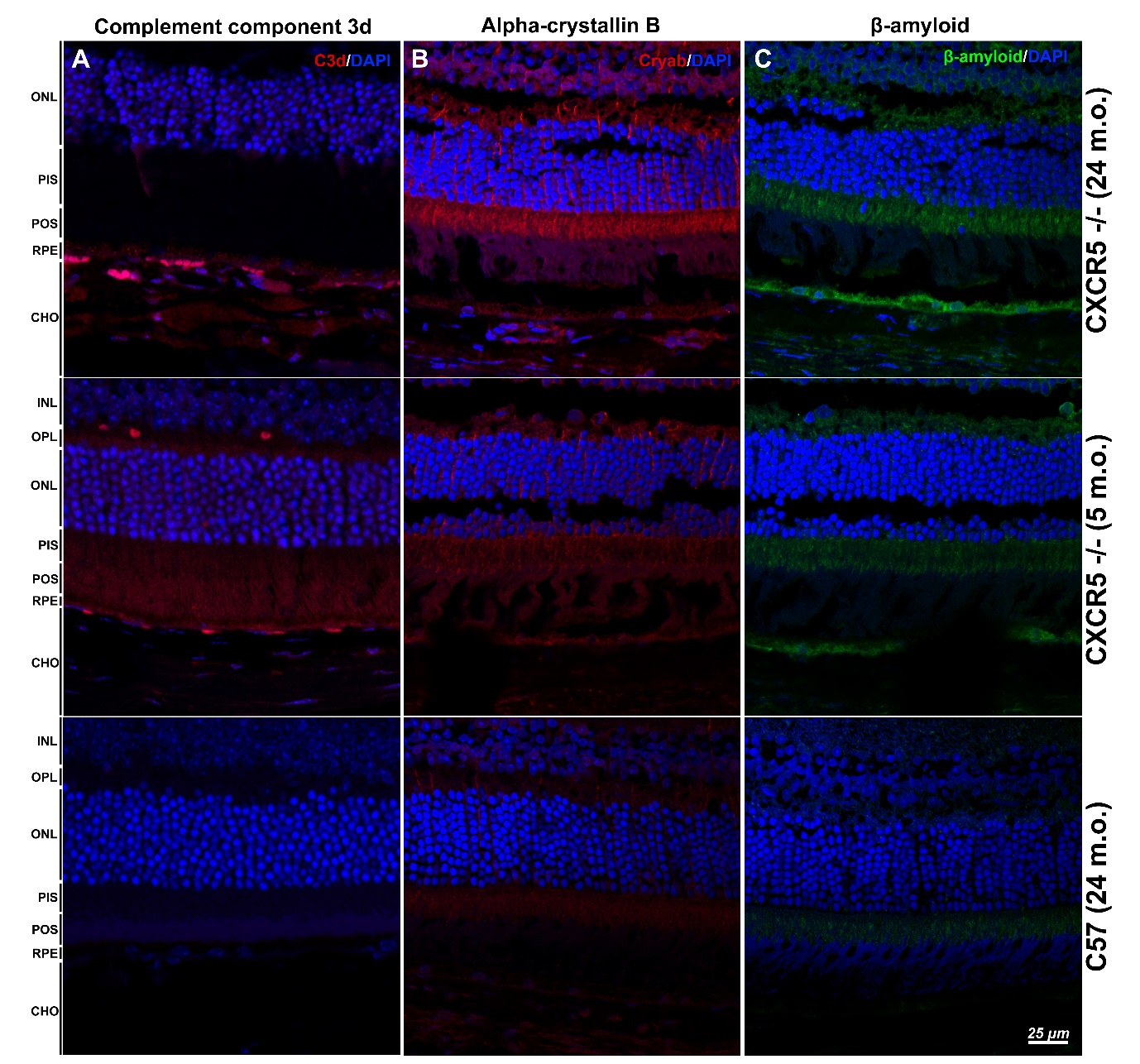


**Supplementary Figure 1.** **Immunofluorescent analysis of AMD-associated proteins.**
(**A**) Complement component 3d (red); (**B**) Alpha-crystallin B (red); (**C**) Amyloid beta (green) staining in mouse eye sections. Nuclear counterstaining by DAPI (blue) in fluorescent images. Samples were obtained from 24, 5 m.o. CXCR5^-/-^ and 24 m.o. C57 control female mice. Retinal layer designations: INL–inner nuclear layer, OPL–outer plexiform layer, ONL–outer nuclear layer, PIS–photoreceptors inner segments, POS–photoreceptors outer segments, RPE–retinal pigment epithelia, and CHO–choroid.


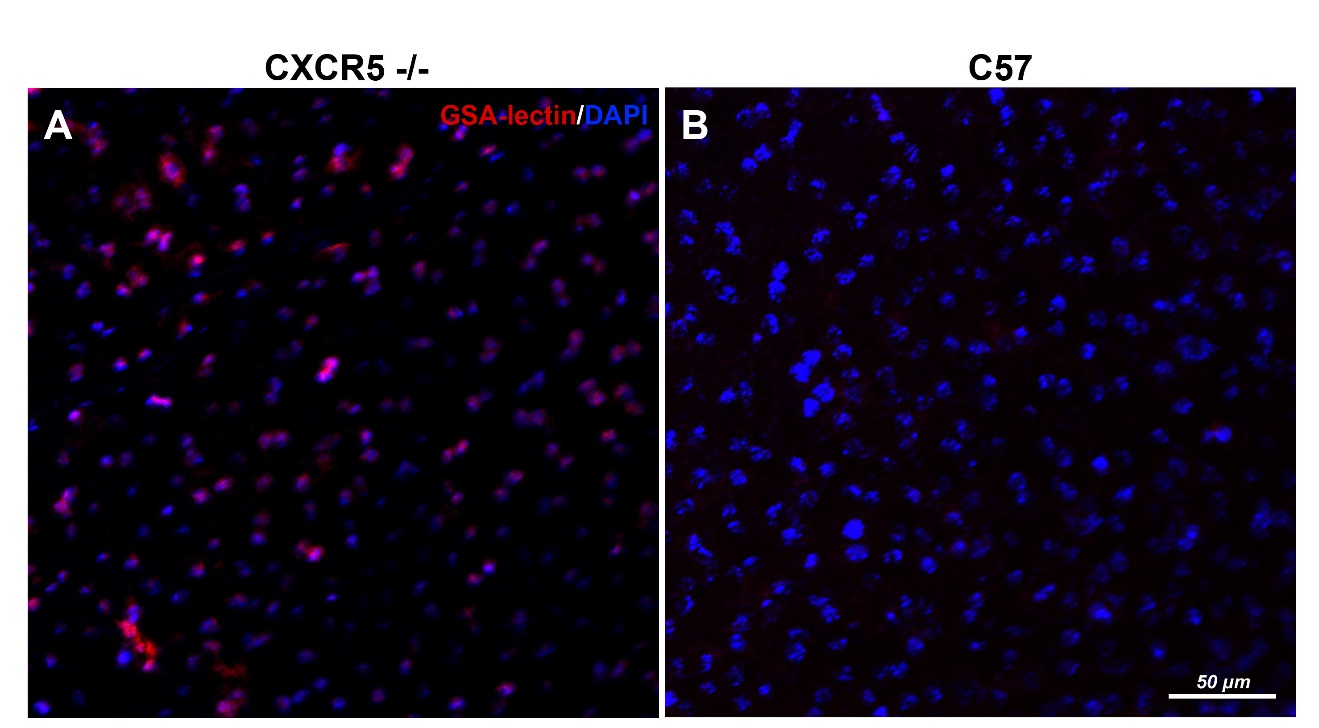


**Supplementary Figure 2.** **Griffonia simplicifolia lectin (GSA) staining (red) of RPE/choroid complexes.** (**A**) The presence of marked GSA-lectin-positive cell infiltration in RPE/choroid complexes of CXCR5^-/-^ mice; (**B**) No GSA lectin signals are detected in C57 RPE/choroid. Samples were obtained from 24 m.o. female mice.

**
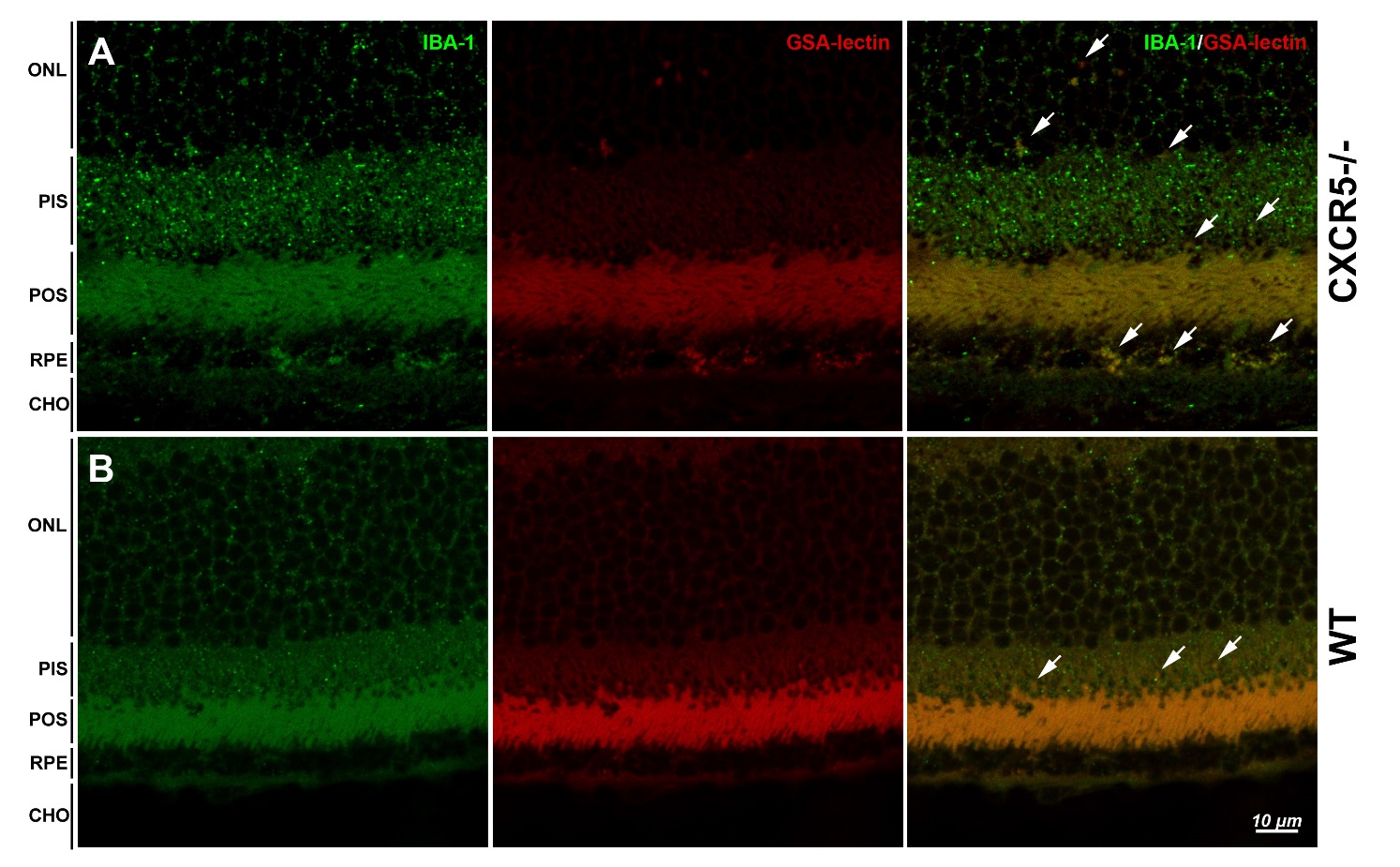
**

**Supplementary Figure 3.** **IBA-1 (green) and Griffonia simplicifolia lectin (GSA) staining (red) of CXCR5 and WT histological sections.** **(A)** Sections from CXCR5^-/-^ mice indicate multiple double-positive signals in sub-retinal space; **(B)** Few double positive cells identified in the retina of WT controls with no sub-retinal infiltration. Arrows indicate the double-labeled cells in the merged images. Samples were obtained from 24 m.o. CXCR5^-/-^ and age-matched WT control female mice. Retinal layer designations: ONL–outer nuclear layer, PIS–photoreceptors inner segments, POS–photoreceptors outer segments, RPE–retinal pigment epithelia, and CHO–choroid.


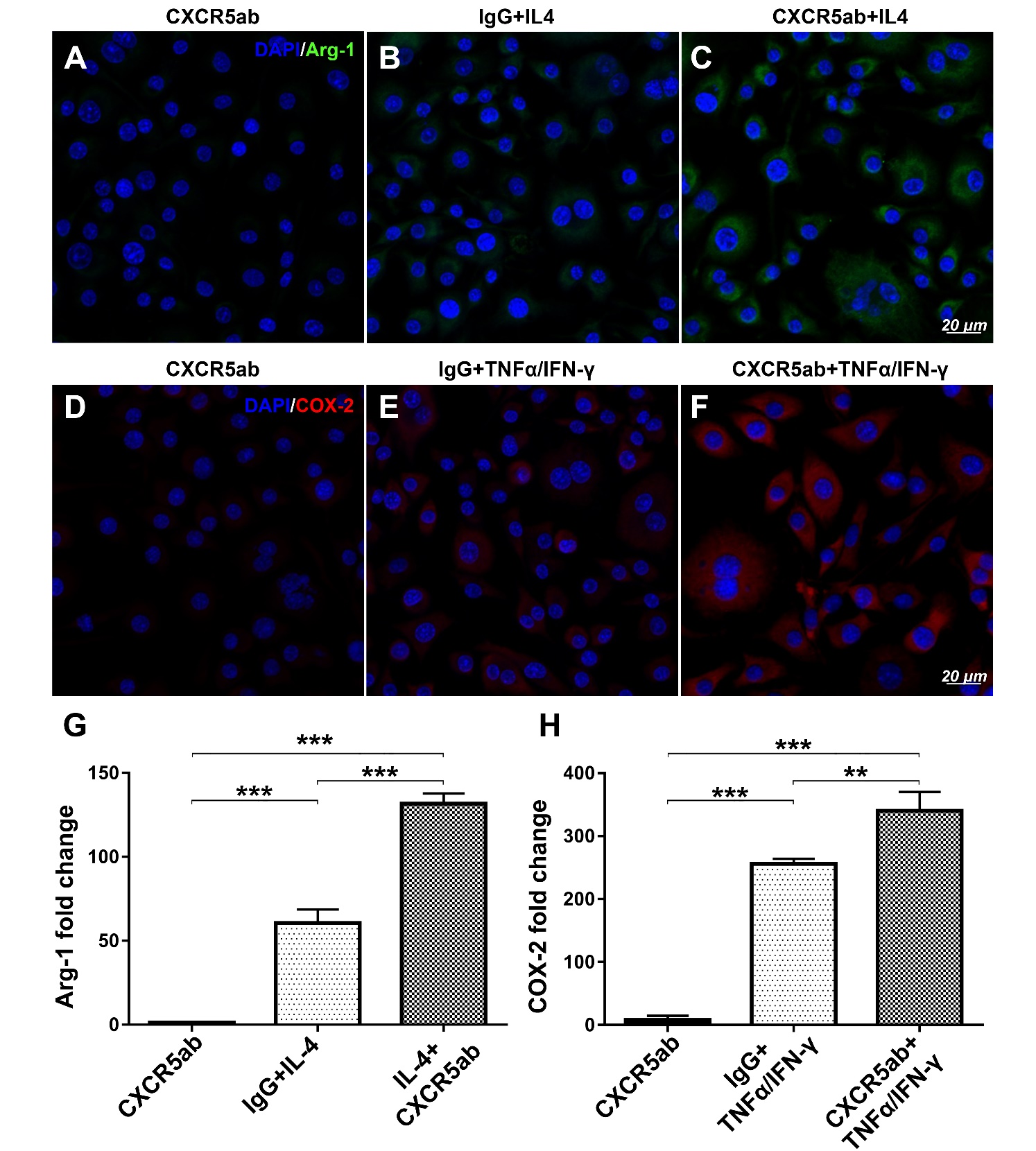


**Supplementary Figure 4. In vitro response of BV-2 cells activation when treated with CXCR5 antibodies. The effects of CXCR5 blockade on Arg-1 and COX-2 expression in BV-2 cells under IL-4 and rmTNFα/rmIFNγ stimulation.** (**A**) Immunofluorescence images of Arg1 (green) pretreated with CXCR5 antibody alone; (**B**) Positive control of IgG pretreatment, stimulated with recombinant mouse interleukin 4 (rmIL-4); **(C)** CXCR5 antibody pretreatment with rmIL-4 stimulation; **(D)** COX-2 (red) pretreated with CXCR5 antibody alone; **(E)** Positive control of IgG pretreatment, stimulated with recombinant mouse rmTNFα/rmIFNγ; (**F**) CXCR5 antibody pretreatment with rmTNFα/rmIFNγ stimulation. The cell nuclei were visualized with DAPI staining (blue); **(G)** Quantitative qRT-PCR analysis of Arg-1; and **(H)** COX-2 expression. (n = 3). A one-way ANOVA test with Tukey multiple comparisons was used to determine statistical significance. **p < 0.01; ***p < 0.001


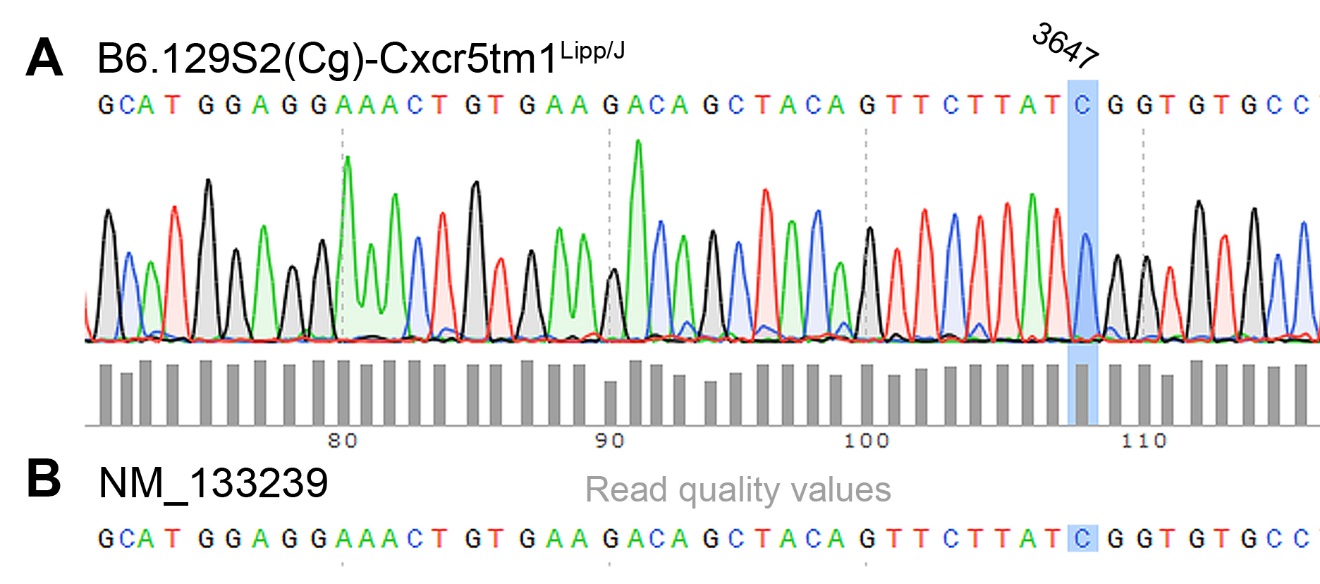


**Supplementary Figure 5. Sanger sequencing identification of CRB1-RD8 mutation in CXCR5-/- animals. (A)** Representative fragment of the CRB-1 Sanger sequence from CXCR5-/- mouse; **(B)** Aligned with the canonical sequence of CRB-1 gene NM_133239. Grey bars indicate read quality values.
